# Identification of suitable reference genes for real-time quantitative PCR analysis of hydrogen peroxide-treated human umbilical vein endothelial cells

**DOI:** 10.1186/s12867-017-0086-z

**Published:** 2017-04-05

**Authors:** Tianyi Li, Hongying Diao, Lei Zhao, Yue Xing, Jichang Zhang, Ning Liu, Youyou Yan, Xin Tian, Wei Sun, Bin Liu

**Affiliations:** Department of Cardiology, The Second Hospital of Jilin University, Changchun, 130041 Jilin China

**Keywords:** Hydrogen peraoxide, Human umbilical vein endothelial cells, qRT-PCR, Reference genes, Normalization

## Abstract

**Background:**

Oxidative stress can induce cell injury in vascular endothelial cells, which is the initial event in the development of atherosclerosis. Although quantitative real-time polymerase chain reaction (qRT-PCR) has been widely used in gene expression studies in oxidative stress injuries, using carefully validated reference genes has not received sufficient attention in related studies. The objective of this study, therefore, was to select a set of stably expressed reference genes for use in qRT-PCR normalization in oxidative stress injuries in human umbilical vein endothelial cells (HUVECs) induced by hydrogen peroxide (H_2_O_2_).

**Results:**

Using geNorm analysis, we found that five stably expressed reference genes were sufficient for normalization in qRT-PCR analysis in HUVECs treated with H_2_O_2_. Genes with the most stable expression according to geNorm were *U6*, *TFRC, RPLP0, GAPDH,* and *ACTB*, and according to NormFinder were *ALAS1, TFRC, U6, GAPDH*, and *ACTB*.

**Conclusion:**

Taken together, our study demonstrated that the expression stability of reference genes may differ according to the statistical program used. *U6, TFRC, RPLP0, GAPDH*, and ACTB was the optimal set of reference genes for studies on gene expression performed by qRT-PCR assays in HUVECs under oxidative stress study.

## Background

Atherosclerosis (AS) is the leading cause of coronary heart disease, and is associated with high morbidity and mortality [1]. The initial event in its development is vascular endothelial injury induced by oxidative stress, which is associated with changes in gene expression [2, 3]. Gene expression studies are therefore of great importance to oxidative stress injury research. Under pathological conditions, such as ischemia–reperfusion and inflammation, reactive oxygen species (ROS) are generated and lead to vascular endothelial injury [4]. As one of the most common ROS, hydrogen peroxide (H_2_O_2_) causes cell and tissue damage through producing the highly reactive radical OH [5, 6]. Thus, H_2_O_2_ has been extensively used as an oxidative stimulus to induce oxidative stress in in vitro models [7].

Analysis of gene expression under different physiological and pathological conditions, including oxidative stress, often uses quantitative real-time polymerase chain reaction (qRT-PCR) because of its low template input requirement, high sensitivity, and high specificity [8, 9]. Given that the expression of target genes is normalized to one or more reference genes in this approach, it is of great importance to use an optimal normalizer for improving the accuracy and reliability of expression measurements [10]. However, this assumes that expression of the reference gene remains constant in all cell/tissue types under specific experimental conditions. Unfortunately, increasing data have shown that no single gene is expressed constantly across all cell types or under all physiological/pathological conditions [11–13]. Therefore, to obtain accurate gene expression information, it is imperative that stable reference genes be chosen for the specific type of tissue and experimental condition [14]. GeNorm [13] and NormFinder [15] are the most commonly used methods to evaluate reference genes, but different statistical algorithms are known to cause inconsistent rankings. Candidate genes can be used as reference genes for the normalization of qRT-PCR results if they demonstrate stable expression under different experimental conditions and statistical algorithms [16].

In this study, 15 common reference genes were identified in HUVECs exposed to different concentrations of H_2_O_2_. GeNorm and NormFinder software was used to calculate the variability of candidate gene expression and to obtain the most suitable reference genes. This study provides a basis for the selection of reference genes and useful guidelines for future gene expression studies in human umbilical vein endothelial cells HUVECs exposed to H_2_O_2_.

## Methods

### Cell culture and H_2_O_2_ studies

HUVECs were purchased from the China Center for Type Culture Collection (Wuhan, China) and cultured in a humidified 5% CO_2_, 37 °C incubator. The vascular cell basal medium (ATCC, USA) added with the endothelial cell growth kit-BBE (ATCC, USA) was used as the complete growth medium for this cell line, and contained the following components: 0.2% bovine brain extract; 5 ng/mL rh EGF; 10 mmol/L l-glutamine; 0.75 units/mL heparin sulfate; 1 µg/mL hydrocortisone hemisuccinate; 2% fetal bovine serum, and 50 µg/mL ascorbic acid. HUVECs were cultured with different concentrations of H_2_O_2_ (0, 500, 1000, 2000, 3000, 4000, 5000, or 6000 µmol/L) for 24 h. Each experiment was performed in triplicate.

### Total RNA extraction

Total RNA from HUVECs was extracted using the Eastep^®^ Super Total RNA Extraction Kit (Promega, USA) following the manufacturer’s instructions. Genomic DNA was eliminated by on-column treatment with RNase-free DNase I. The concentration and purity of RNA were measured using a NanoDrop 2000 spectrophotometer (Thermo, USA).

### Reverse transcription

Purified RNA was reverse transcribed immediately after extraction with the TranScript One-Step gDNA Removal and cDNA Synthesis SuperMix Kit (Transgen Biotech, China) according to the manufacturer’s instructions. For each sample, cDNA was synthesized from 300 ng total RNA in a final volume of 20 µL and stored at −20 °C until further use.

### Quantitative real-time PCR

All primers were purchased from Sangon Biotech, China. Primer sequences are listed in Table 1. qRT-PCR was performed in 96-well plates using the Light Cycler 480 system (Roche, Swiss). Each 20-µL reaction contained 10 µL of TransStart Green qPCR SuperMix (Transgen Biotech, China), 0.5 µL of each primer (10 μmmol/L), 8 µL of ddH_2_O, and 1 µL of cDNA. PCR conditions were as follows: 95 °C for 1 min, followed by 40 cycles at 95 °C for 20 s, and 61 °C for 31 s.

**Table 1:** Name, primer sequences, and product size of candidate reference genes

Symbol	Gene name	Primer sequences (forward/reverse)	Product length (bp)
*18S*	18S ribosomal RNA	CGGCTACCACATCCAAGGAA/GCTGGAATTACCGCGGCT	186
*GAPDH*	Glyceraldehyde-3-phosphate dehydrogenase	GACAGTCAGCCGCATCTTCT/TTAAAAGCAGCCCTGGTGAC	120
*U6*	U6 snRNA	AACGCTTCACGAATTTGCGT/CTCGCTTCGGCAGCACA	109
*ALAS1*	5′-Aminolevulinate synthase 1	GGCAGCACAGATGAATCAGA/CCTCCATCGGTTTTCACACT	150
*ACTB*	Actin, beta	AGAAAATCTGGCACCACACC/TAGCACAGCCTGGATAGCAA	173
*TFRC*	Transferrin receptor	GTCGCTGGTCAGTTCGTGATT/AGCAGTTGGCTGTTGTACCTCTC	80
*PPIA*	Peptidylprolyl isomerase A	AGACAAGGTCCCAAAGAC/ACCACCCTGACACATAAA	118
*RPLP0*	Ribosomal protein lateral stalk subunit P0	CCATTCTATCATCAACGGGTACAA/TCAGCAAGTGGGAAGGTGTAAT	75
*PBGD*	Hydroxymethylbilane synthase	AGTGTGGTGGGAACCAGC/CAGGATGATGGCACTGAACTC	144
*GUSB*	Glucuronidase beta	AGCCAGTTCCTCATCAATGG/GGTAGTGGCTGGTACGGAAA	160
*B2M*	Beta-2-microglobulin	AGCGTACTCCAAAGATTCAGGTT/ATGATGCTGCTTACATGTCTCGAT	206
*HPRT1*	Hypoxanthine phosphoribosyl transferase 1	GACCAGTCAACAGGGGACAT/CCTGACCAAGGAAAGCAAAG	132
*RPL29*	Ribosomal protein L29	GGCGTTGTTGACCCTATTTC/GTGTGTGGTGTGGTTCTTGG	120
*PUM1*	Pumilio RNA binding family member 1	CAGGCTGCCTACCAACTCAT/GTTCCCGAACCATCTCATTC	211
*TBP*	TATA-box binding protein	TGCACAGGAGCCAAGAGTGAA/CACATCACAGCTCCCCACCA	132

### Statistical analysis

Two versions of Excel-based software, geNorm and NormFinder, were used to evaluate the stability of the candidate reference genes. For both versions, Ct values were converted to relative quantities for analysis according to the formula: $2^{{ - \Delta {\text{Ct}}}} \left( {\Delta {\text{Ct}} = {\text{corresponding Ct value}}-{\text{minimum Ct}}} \right)$ [17].

GeNorm software analyzes gene stability based on the average pairwise variation of a particular gene against all other control genes as the M value. Candidate reference genes are ranked according to their expression stability by stepwise exclusion of genes with the highest M-value. Genes with the lowest M value have the most stable expression [13]. To determine the possible need or utility of control genes for normalization, the pairwise variation V_n/n+1_ was calculated between the two sequential normalization factors NF_n_ and NF_n+1_. For the pairwise variation V_n/n+1_, 0.2 was taken as a cut-off value, below which the inclusion of an additional control gene is not required [13, 18].

NormFinder software, based on an ANOVA mathematical model, estimates both intra- and inter-group expression variation and calculates a candidate gene stability value. A lower stability value indicates a more stable reference gene expression [15].

## Results

### Expression profiles of candidate reference genes

**Fig. 1:**
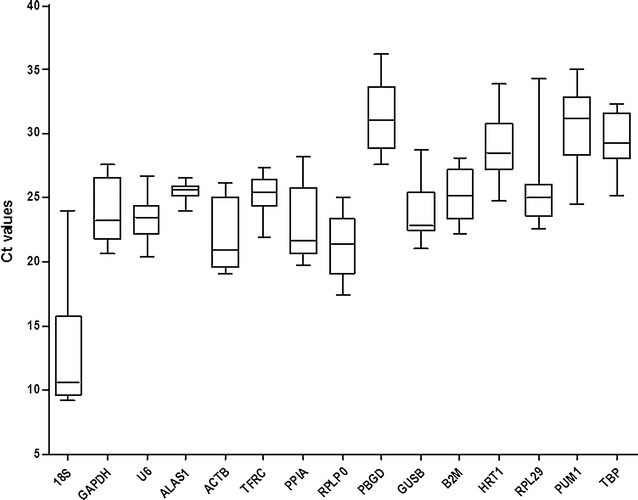
Ct values of 15 candidate reference genes in all samples. Raw Ct values of eight samples, under normal conditions and different concentrations of H_2_O_2_, are described using a *box* and *whisker plot*. The *outer box* is determined from the 25th to the 75th percentiles, and the *line* across the box is the median

Fifteen candidate reference genes in HUVECs were analyzed by qRT-PCR (Table 1), and their Ct values are shown in Fig. 1. The Ct values ranged from 9.225 to 36.19, representing a wide variation, although most were in the range of 22–27. The most highly expressed gene was *18S*, which exhibited a median Ct value of 10.66. All other genes had median Ct values larger than 20, while *PUM1* presented with the lowest expression level with a median Ct value of 31.2. *18S* had the widest range of 14.83 cycles, whereas *ALAS1* had the narrowest range of 2.665 cycles. Mean Ct, STDEVP (STD), and coefficient of variation (CV) were calculated as shown in Table 2. CV values for candidate reference genes ranged from 2.89 to 36.64%. *ALAS1* had the lowest CV, at 2.89%, indicating the lowest variation in gene expression. By contrast, the CV of *18S* was the highest at 36.64%, indicating the highest variation in gene expression.

**Table 2:** Mean Ct, STD, and coefficient of variation of candidate reference genes

Gene name	Mean Ct	STD	CV (%)
18S	13.01	4.77	36.64
GAPDH	23.88	2.36	9.89
U6	23.4	1.76	7.53
ALAS1	25.57	0.74	2.89
ACTB	21.9	2.62	11.96
TFRC	25.24	1.58	6.27
PPIA	22.87	2.85	12.46
RPLP0	21.38	2.37	11.06
PBGD	31.29	2.68	8.56
GUSB	23.86	2.3	9.62
B2M	25.2	1.95	7.75
HRT1	28.97	2.54	8.78
RPL29	25.81	3.37	13.04
PUM1	30.52	3.05	10
TBP	29.38	2.14	7.28

### Expression stability of candidate reference genes

#### GeNorm analysis

**Fig. 2:**
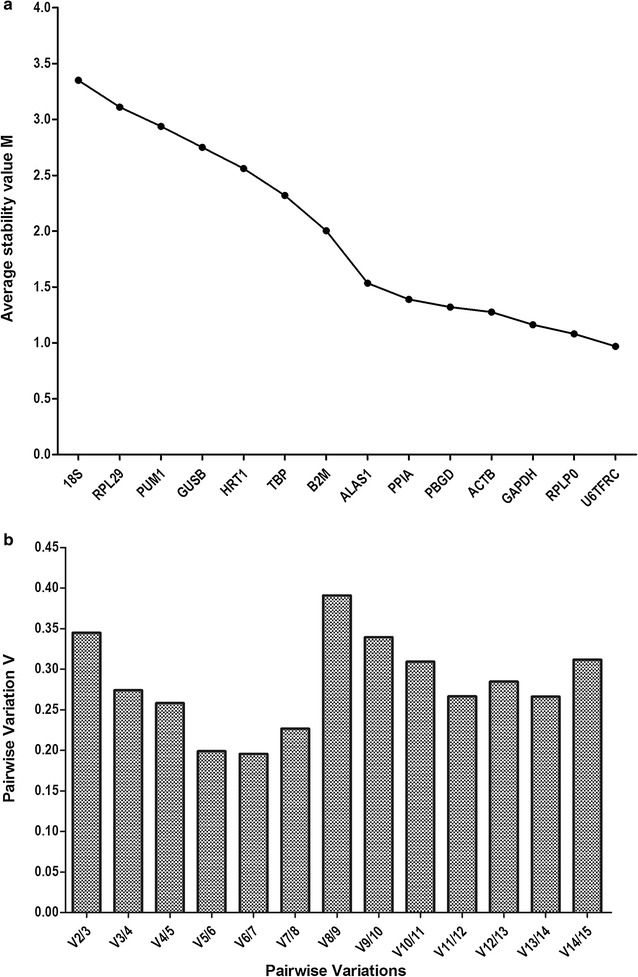
Expression stability values of reference genes analyzed by geNorm software. **a** Average expression stability measures (M) of reference genes. The x-axis from *left to right* indicates the ranking of the genes according to their stability; higher M values indicate lower stabilities. **b** Determination of the suitable number of reference genes required for normalizing. The software calculates the normalization factor from at least two genes, and the V value defines the pair-wise variation between two sequential normalization factors

GeNorm software was used to evaluate the stability of candidate reference genes, and the calculated M values are shown in Fig. 2a. A lower M value indicates a higher stability. *U6* and *TFRC* had the lowest M values of 0.97, whereas *18S* had the highest M value of 3.35. The three reference genes of highest stability were *U6*, *TFRC*, and *RPLP0*, while *18S*, *RPL29*, and *PUM1* showed the lowest stability. Figure 2b shows the pairwise variation for all data. *V5/V6* was found to be lower than 0.2, suggesting that the top five reference genes were adequate for normalization, and that an additional 16 reference gene was not necessary.

#### NormFinder analysis

**Fig. 3:**
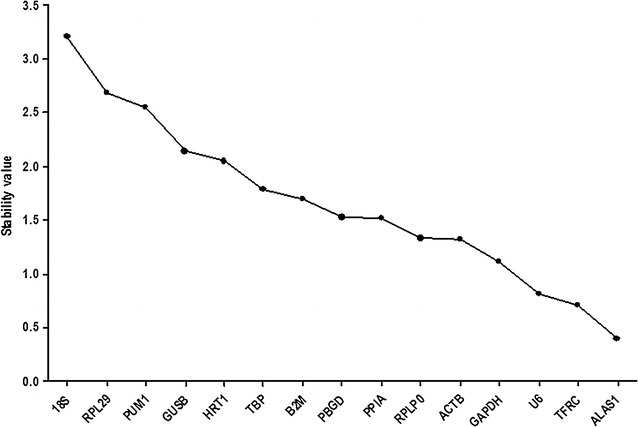
Stability values of each reference gene from the NormFinder algorithm. Ranking of candidate reference genes based on stability values calculated by NormFinder

The expression stability of candidate reference genes was also calculated using NormFinder software. Similar to the GeNorm software, genes with the lowest stability value are the most stable expressed ones. As shown in Fig. 3, the most stable reference gene was *ALAS1*, following *TFRC* and *U6*. The least stable reference genes were *18S*, *RPL29*, and *PUM1*. A list of candidate reference genes ranked according to stability by the two versions of software is shown in Table 3.

**Table 3:** Ranking of candidate reference genes evaluated by geNorm and NormFinder statistical algorithms

Gene name	geNorm		NormFinder	
	Stability value	Rank	Stability value	Rank
U6	0.97	1	0.81	3
TFRC	0.97	2	0.71	2
RPLP0	1.08	3	1.33	6
GAPDH	1.16	4	1.11	4
ACTB	1.28	5	1.32	5
PBGD	1.32	6	1.52	8
PPIA	1.39	7	1.52	7
ALAS1	1.54	8	0.4	1
B2M	2	9	1.7	9
TBP	2.32	10	1.79	10
HRT1	2.56	11	2.05	11
GUSB	2.75	12	2.14	12
PUM1	2.94	13	2.55	13
RPL29	3.11	14	2.69	14
18S	3.35	15	3.21	15

## Discussion

ROS are involved in the pathophysiology of cardiovascular diseases such as hyperlipidemia, hypertension, ischemic heart disease, and chronic heart failure [19, 20]. They also cause changes in gene expression, which can be accurately and sensitively measured by qRT-PCR [17, 20]. This technique normalizes the gene of interest against an endogenous control whose expression remains unaltered in the samples under analysis [21]. The concept of validating reference genes used for normalization in qRT-PCR analysis before use was initially suggested in 2002 [22], and has been realized in various scientific disciplines such as plant sciences [23, 24], cancer [25, 26], stem cells [27, 28], and cardiovascular research [14, 29, 30]. Considering that an algorithm is one-sided for evaluating the expression stability of reference genes, many statistical approaches are usually integrated to determine the optimal reference genes under different experimental conditions [17].

The Ct value is used to evaluate gene expression in qRT-PCR analysis. At the same RNA concentration, gene expression levels are negatively associated with Ct values [31, 32]. Generally, neither a very high (threshold cycle Ct > 30) Ct value of a reference gene nor a very low (Ct < 15) is suitable for qRT-PCR [32]. In the present study, the Ct values of the 15 candidate reference genes tested showed large variations across all of the tested samples. The Ct values of *PBGD*, *HRT1*, *RPL29*, *PUM1*, and *TBP* in some samples were higher than the threshold value, whereas that of *18S* in some samples was lower than the threshold value. Therefore, these six genes should not be used as reference genes in HUVECs treated with H_2_O_2_. The Ct value of the remaining candidate reference genes, *GAPDH*, *ALAS1*, *U6*, *TFRC*, *ACTB*, *PPIA*, *RPLP0*, *GUSB*, and *B2M*, ranged from 15 to 30. This analysis indicates that the most suitable reference genes in HUVECs treated with H_2_O_2_ should be selected from this list.

CV values can represent the variability of candidate reference genes and reflect their stability to some extent. However, analysis according to CV alone is not sufficiently reliable. In our study, the CV value of *TBP* is relatively low, indicating a low variation in gene expression. However, according to geNorm and NormFinder software analysis, *TBP* stability is relatively low. This finding demonstrates the importance of evaluating the stability of reference genes for the normalization of gene expression under different experimental treatments.

Notably, the stability of some reference genes may vary under different conditions. 18S and GAPDH have been widely used as the reference for gene analysis in qRT-PCR [33]. However, our data showed that 18S was the least stable reference gene and GAPDH was not the best choice for gene analysis in HUVECs under H_2_O_2_ treatment. ACTB was reported to be unstable in HUVECs in response to hypoxia [34], but was stably expressed in HUVECs treated with H_2_O_2_ in the present study. However, RPLP0 and TFRC were reported to be the most stably expressed reference genes in HUVECs treated with hypoxia [34], which was confirmed by our present findings. These findings demonstrated that studying the expression stability of reference genes under different conditions was important for gene expression research.

The ranking for expression stability of reference genes may differ according to the statistical program used. In the present study, we employed two different statistical programs, geNorm and NormFinder, to evaluate gene expression stability in HUVECs treated with H_2_O_2_. The majority of the results from both versions of software were the same. For example, following both analyses, the rank of *TFRC*, *GAPDH*, and *ACTB* was shown to be relatively high, while the least stable genes were *18S*, *RPL29*, *PUM1*, *GUSB*, *HRT1*, *TBP*, and *B2M*. However, the results from the two software versions showed some differences, notably the ranks of *U6* and *RPLP0* were different though both relatively high. Considering this fact, it appears that *U6* and *RPLP0* are relatively stable in HUVECs exposed to H_2_O_2_. However, though NormFinder analysis suggested that *ALAS1* is the most stable reference gene, this was not confirmed by geNorm software which calculated M > 1.5. In this case, we considered that *ALAS1* is not a reliable reference gene in HUVECs treated with H_2_O_2_. Some previous reports have indicated that a single gene is not a reliable reference for normalization [13]. For this reason, we propose using a combination of five stably expressed reference genes (*U6*, *TFRC*, *RPLP0*, *GAPDH*, and *ACTB*).

In addition, we used extracellular H_2_O_2_ as an oxidative stress model, which has been employed worldwide. Besides extracellular H_2_O_2_, there are some other models for oxidative stress induction, including extracellular O_2_^−^ and normobaric hyperoxia [35]. The toxic effects of extracellular O_2_^−^ and H_2_O_2_ are almost similar; still, there are some differences between these two models. On the one hand, H_2_O_2_ crosses cellular membrane easily, which is an advantage over extracellular O_2_^−^; on the other hand, the effect of H_2_O_2_ depends on the cell density. Hyperoxia is a relative measure, defined as oxygen concentration higher than normal. Reactive oxygen species in hyperoxia model are generated intracellular, which is different from extracellular H_2_O_2_ and O_2_^−^.

Actually, measurement of a suitable positive control could help to validate these reference genes. However, despite validation, the positive control has no significant influence on the stability evaluation by the calculation software. Thus, many studies [14, 36–39] evaluated suitable reference genes by calculation software without the positive control.

## Conclusions

Our study demonstrates that a combination of *U6*, *TFRC*, *RPLP0*, *GAPDH*, and *ACTB* is the optimal reference gene set for HUVECs treated with H_2_O_2_. These will be useful for studies on gene expression in response to oxidative stress induced by ROS in HUVECs.

## Abbreviations

qRT-PCR: quantitative real-time polymerase chain reaction
HUVEC: human umbilical vein endothelial cell
H_2_O_2_: hydrogen peroxide
AS: atherosclerosis
ROS: reactive oxygen species
STD: STDEVP
CV: coefficient of variation
18S: 18S ribosomal RNA
GAPDH: glyceraldehyde-3-phosphate dehydrogenase
U6: U6 snRNA
ALAS1: 5′-aminolevulinate synthase 1
ACTB: actin, beta
TFRC: transferrin receptor
PPIA: peptidylprolyl isomerase A
RPLP0: ribosomal protein lateral stalk subunit P0
PBGD: hydroxymethylbilane synthase
GUSB: glucuronidase beta
B2M: beta-2-microglobulin
HPRT1: hypoxanthine phosphoribosyl transferase 1
RPL29: ribosomal protein L29
PUM1: pumilio RNA binding family member 1
TBP: TATA-box binding protein
